# Radiological Image-Guided Placement of Covered Niti-S Stent for Palliation of Dysphagia in Patients with Cervical Esophageal Cancer

**DOI:** 10.1007/s00455-013-9446-0

**Published:** 2013-01-31

**Authors:** Takeshi Fujita, Masahiro Tanabe, Kensaku Shimizu, Etsushi Iida, Naofumi Matsunaga

**Affiliations:** 1 Department of Radiology, Ube Industries, Ltd. Central Hospital, 750 Nishikiwa, Yamaguchi Ube, 755-0151 Japan; 2 Department of Radiology, Yamaguchi University Graduate School of Medicine, 1-1-1 Minamikogushi, Yamaguchi Ube, 755-8505 Japan

**Keywords:** Cervical esophageal cancer, Covered Niti-S stent, Dysphagia, Palliative care, Radiological image guidance, Deglutition, Deglutition disorders

## Abstract

The aim of this study was to evaluate the clinical effectiveness of covered Niti-S stent placement under multidetector CT and fluoroscopy guidance for the palliation of dysphagia in patients with cervical esophageal cancer. Under radiological imaging guidance using axial and sagittal CT scans, and fluoroscopy, Niti-S esophageal stents were placed in ten consecutive patients with complete obstruction caused by cervical esophageal cancer (9 men and 1 woman; age range = 54–79 years; mean age = 68.1 years) between February 2010 and December 2011. The procedure time and technical success rate were evaluated. Swallowing improvement was assessed by the following items: ability to eat and/or swallow (graded as follows: 3 = ability to eat normal diet, 2 = ability to eat semisolids, 1 = ability to swallow liquids, 0 = complete obstruction). Procedural and post-procedural complications were also evaluated. Survival (mean ± SD) was examined. The mean (±SD) procedure time was 40 ± 19 min (range = 21–69 min). Stent placement was technically successful in all patients; inadequate stent deployment did not occur in any case. Ability to eat and/or swallow was improved and scored 2.4 (score 3 in 5 cases, score 2 in 4 cases, score 1 in 1 case, and score 0 in no case) after stent placement. No major or post-procedural complications were encountered. The mean survival time was 131 ± 77 days (range = 31–259 days). Niti-S stents appeared to be a safe and effective device for the palliation of dysphagia caused by advanced cervical esophageal cancer. Multidetector CT and fluoroscopy image guidance helped the operators accurately place the stents in the cervical esophagus.

## Introduction

Malignant esophageal obstruction is caused by a primary esophageal neoplasm in most patients [1]. Unfortunately, despite recent advances in the curative treatment of esophageal cancer, including combination chemotherapy and radiotherapy, many patients present with the disease at an incurable stage, requiring palliative treatment to relieve dysphagia, which is often their main symptom [2, 3].

Self-expandable metal stents (SEMSs) were developed with the advantage of having a smaller, more flexible delivery system and increased ease of deployment [4]. Currently, covered stents are the most commonly used SEMSs in patients with esophageal cancer because they restrict tumor ingrowth through the metal mesh [5]. Covered SEMSs also have been used successfully in the management of patients with anastomotic leaks or fistulas [6]. Despite the large number of different covered SEMSs available on the market, nitinol stents are widely used [7]. One of the most commonly used nitinol-covered SEMSs worldwide is the partially covered Ultraflex stent (Boston Scientific Japan, Tokyo, Japan), because it is very flexible and exerts less radial force; it is thus recommended to decrease the risk of pain associated with the use of the stiffer devices [8–10].

It is essential to place the stent in the correct position for it to be effective. It is very important to place the covered SEMS correctly in a patient with cervical esophageal cancer, because if placed improperly, the patient may experience a troublesome foreign body sensation in the cervical region or serious swallowing disturbance when the proximal end of the covered SEMS extends into the upper esophageal orifice or hypopharynx [11, 12]. Some studies reported that the placement of a conventional stent has been relatively contraindicated in this region [12, 13]. The newly designed covered Niti-S stent is fully covered to resist tissue ingrowth, and it has an outer nitinol wire that reduces the risk of stent migration. This covered SEMS does not become shortened at the proximal end when it is deployed so it is relatively easy to adjust its position [14, 15].

In the current study, Niti-S esophageal covered stents were placed under multidetector CT and fluoroscopic guidance to achieve precise placement, and the safety and clinical effectiveness of the covered Niti-S stent for the palliation of dysphagia in patients with cervical esophageal cancer were determined.

## Materials and Methods

### Patient Population

The institutional review board approved this study. Written informed consent was obtained from the patients for all procedures. Ten consecutive patients with complete dysphagia caused by advanced cervical esophageal cancer (9 men and 1 woman; age range = 54–79 years; mean age = 68.1 years) were enrolled in this prospective study between February 2010 and December 2011. Squamous cell carcinoma was confirmed pathologically in all patients. The inclusion criteria were an inoperable, advanced malignant obstruction of the cervical esophagus or recurrent dysphagia after prior chemoradiation with curative or palliative intent for esophageal cancer. A tumor was considered inoperable if the patient had distant metastases or local tumor infiltration in neighboring organs and/or poor general condition because of concomitant disease. In two of ten patients, a tracheoesophageal fistula was also present with a cervical esophageal stricture. The upper end of the stricture was located at least 2.0 cm below the upper esophageal orifice on the endoscopic findings in all cases. Exclusion criteria were tumor growth within the upper esophageal orifice or lower hypopharynx, previous stent placement, abnormal coagulation status (an international normalized ratio value >1.5 and a platelet count <70,000 mm^3^), poor general performance status, and unfit to undergo conscious sedation. The primary end points of the study were procedure duration time, technical success rate, complications, and improvement of dysphagia. Secondary outcomes included recurrent dysphagia, which was defined as occurrence of tissue ingrowth or overgrowth, stent migration, and mean survival time.

### Stent Characteristics

**Fig. 1:**
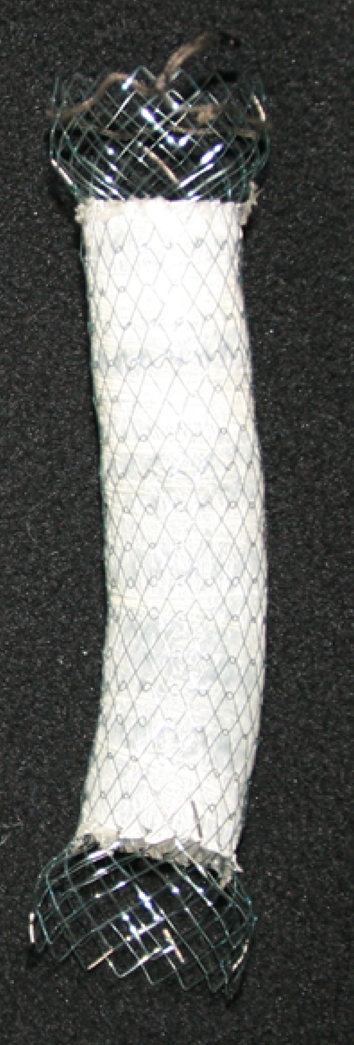
Niti-S-covered esophageal stent

The Niti-S stent (Taewoong Medical, Seoul, Korea) is a compressed form inside an introducer sheath and is characterized by a nitinol monofilament, fine mesh that is fully or partly covered with polyurethane with a proximal flare of 26 mm, a body diameter of 18 mm, and a length of 80, 100, or 120 mm. The proximal end of the stent is flared slightly and has a tulip-like shape with an increase in diameter in order to prevent migration. Moreover, this stent has a double-layer configuration, with an outer uncovered nitinol wire tube to allow the stent to fix itself in the esophageal wall (Fig. 1). It becomes shortened by approximately 35 % from the distal side when it is deployed, whereas the proximal end does not become shortened. The delivery system has a 16-F diameter, which is similar to that of the Ultraflex stent.

### Stent Placement

**Fig. 2:**
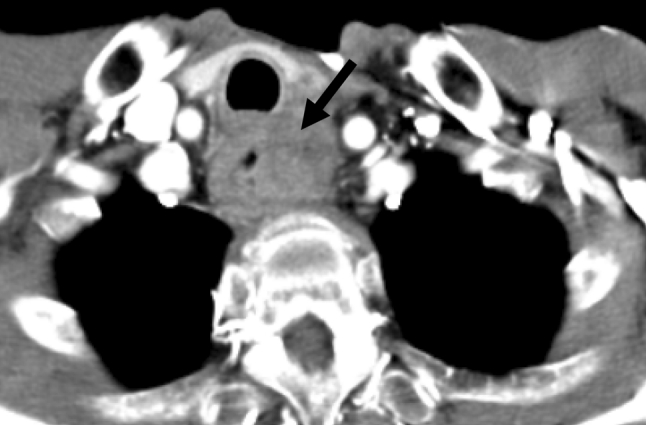
Contrast-enhanced axial CT image a week before the stent placement shows the esophageal cancer (*arrow*) in the cervical region in a 64 year-old man

**Fig. 3:**
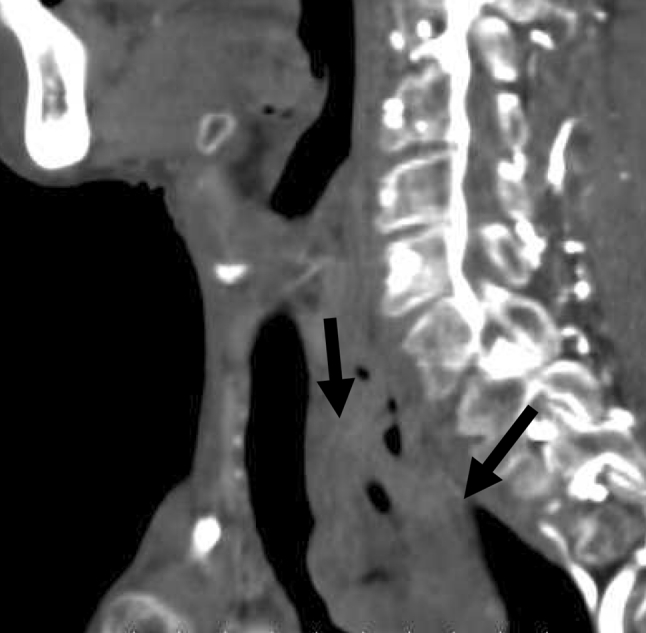
Sagittal CT image obtained with an IVR-CT system immediately before the stent placement demonstrates that cervical esophageal cancer (*arrows*) does not directly invade the hypopharynx

**Fig. 4:**
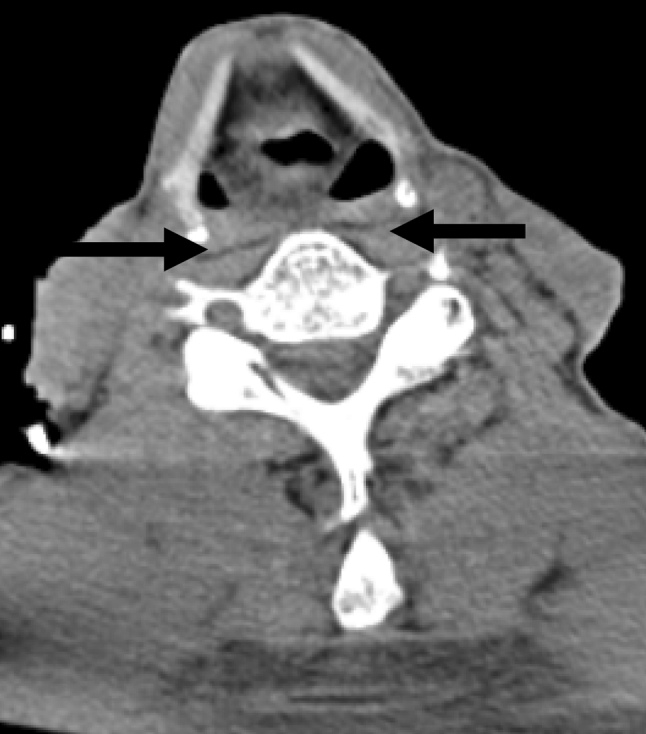
Upper esophageal orifice (*arrows*) is clearly revealed on unenhanced axial CT image

**Fig. 5:**
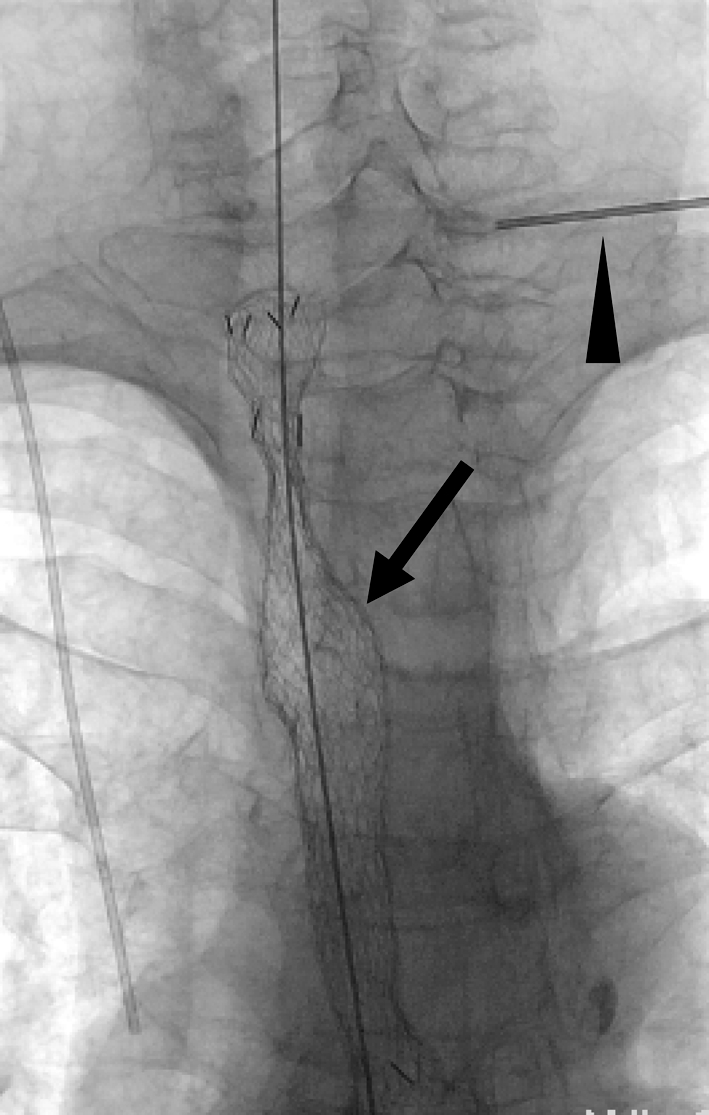
Covered Niti-S esophageal stent is deployed (*arrow*). The radiopaque marker is placed on the body surface as the seventh cervical vertebra (*arrowhead*)

**Fig. 6:**
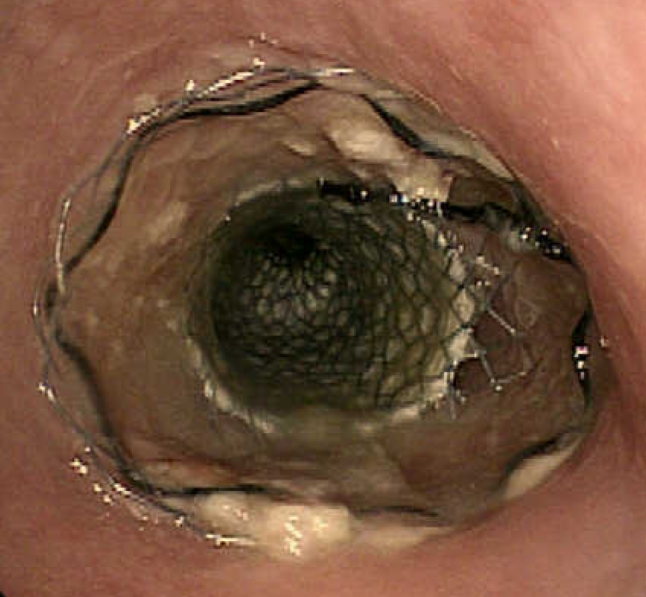
Endoscopic view of expanding Niti-S stent the day after insertion

**Fig. 7:**
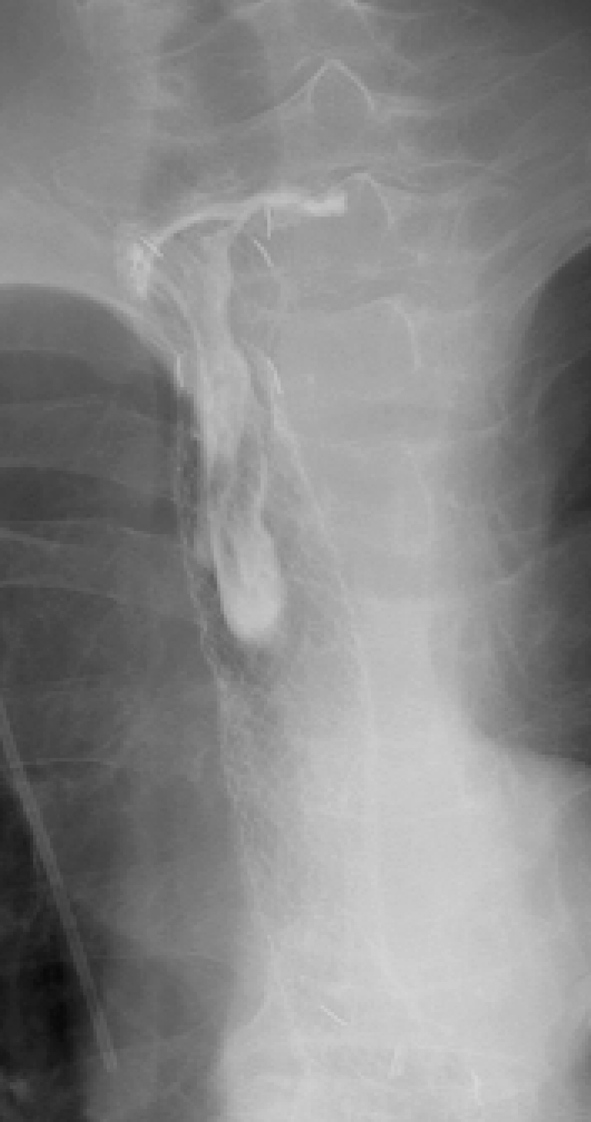
Contrast study shows satisfactory positioning and expansion of the stent 2 days after deployment

All procedures for stent placement were performed by two abdominal interventional radiologists (TF, MT) with 23 and 11 years of experience, respectively. An esophagram using Iopamidol 300 (Iopamiron 300, Bayer Japan, Tokyo, Japan) and conventional contrast-enhanced CT were obtained to delineate the site and length of the cervical stricture and the location of the tumor 3 or 4 days before stent placement (Fig. 2). Barium was not used in patients with a tracheoesophageal fistula because it could cause mediastinitis [16]. The location of the tracheoesophageal fistula was also evaluated, i.e., its size and relationship with the esophageal entrance, and the distance of the most proximal end of the lesion from the incisors was carefully assessed. Esophageal stents were inserted in the Interventional Radiology (IVR)-CT (Somatom Sensation Open, Siemens, Erlangen, Germany) suite under radiologic guidance and conscious sedation without endoscopic assistance. This IVR-CT system consisted of both a multidetector row CT scanner and digital subtraction angiography (DSA), so that both a CT scan and fluorography can be obtained in a single examination session. Pentazocine hydrochloride (Pentagin, Daiichi-Sankyo Healthcare Co., Ltd., Tokyo, Japan) as premedication was injected intramuscularly to provide conscious sedation, and lidocaine hydrochloride (Xylocaine, AstraZeneca K.K., Osaka, Japan) 10 % oral spray was sprayed onto the posterior wall of the oropharynx to reduce the gag reflex with the patient on the table in the supine position. Oxygen (2 L/min) was administered via nasal cannula, and the patient’s vital signs were monitored continuously with pulse oximetry and electrocardiography. The cervical CT examinations were performed with a 20-detector row CT scanner. After acquisition of the topogram, unenhanced axial images were obtained during a breath-hold. The multidetector CT parameters were as follows: detector configuration, 1.2 mm × 20; table feed, 15 mm/rotation; gantry rotation time, 0.7 s. Images were acquired with a 2.0 mm slice thickness and reconstructed into 2.0 mm sections for interpretation. Axial and sagittal multiplanar reconstructions (MPR) were automatically generated to help the operators confirm the exact position of the orifice of the upper esophageal sphincter (= esophageal orifice) and the tumor location (Figs. 3 and 4). Next, radiopaque markers were placed on the body surface to mark the appropriate position where it was planned to place the proximal end of the stent below the upper esophageal orifice by adhesive tape before the procedure. A 150 cm long, 0.035 in. hydrophilic guide wire with a 45° angled, 3.0 cm soft tip (Radifocus guide wire, TERUMO Medical Products, Tokyo, Japan) was carefully inserted into the stomach through the stricture via the oral route under fluoroscopic guidance. Then, the 4-Fr cobra-shaped angiographic catheter was passed into the stomach over the guide wire. After removing the Radifocus guide wire, a 260 cm-long Amplatz stiff guide wire (COOK Japan, Tokyo, Japan) was looped in the stomach or advanced into the proximal duodenum, and the angiographic catheter was exchanged for a 25 cm-long, 6-Fr angiographic sheath (Medikit, Tokyo, Japan). The sheath was inserted into the distal side of the stricture over the long, stiff guide wire. After removing the sheath introducer, leaving the long stiff wire, the location of the tumor was defined by contrast material diluted with normal saline which was injected through the side arm of the sheath above and below the esophageal stricture. A Niti-S covered stent of appropriate size and length was chosen and advanced across the stricture on its delivery system. To prevent migration, it was deployed in such a way that slightly more of the stent was above than below the stricture. The length of the stent was chosen so at least 2 cm of normal esophagus was covered by the stent above and below the stricture. However, the proximal end of the stent was never placed beyond the esophageal orifice under the guidance of the body mark when the stricture was located directly below the esophageal orifice (Fig. 5). Long strictures may require more than one stent with a one-third overlap between stents. After stent deployment, a 6-Fr long sheath with introducer was again inserted over the wire, and contrast material was injected to confirm the correct stent position and rule out any complications such as perforation. An esophagram and gastrointestinal endoscopy were obtained a few days after to show that the stent had adequately expanded in a satisfactory position and that the tracheoesophageal fistula was completely occluded (Figs. 6 and 7). Preballoon dilatation of the stricture was not performed in any case because the stent delivery system could pass the esophageal stricture over the guide wire in all cases. Immediate post-balloon dilatation after stent deployment to avoid the stent migration was also not done in any case.

### Statistical Analysis

The procedure duration time from arrival at the IVR-CT unit to completion of the entire procedure was recorded for all patients. The technical success rate was evaluated after stent placement. Moreover, patients received weekly follow-up physical examinations or telephone calls from 28 days after treatment until death. Assessment of improvement of dysphagia included the following items: ability to eat and/or swallow (graded as follows: 3 = ability to eat a normal diet, 2 = ability to eat semisolids, 1 = ability to swallow liquids, 0 = complete obstruction). Procedural and post-procedural complications were also evaluated. The procedural complications were perforation, aspiration, hemorrhage, stent migration, and pain. Post-procedural complications included perforation, hemorrhage, stent migration, pain or foreign body sensation, and tumor ingrowth or overgrowth. All available charts and records were reviewed in all cases. Survival (mean ± SD) was calculated from the date of stent placement to the date of death.

## Results

The mean procedure time was 40 ± 19 (SD) min (range = 21–69 min). Stent placement was technically successful in all patients, and inadequate stent deployment did not occur in any case. All stents were placed correctly at the planned position, and no stent reached the upper esophageal orifice. One stent was placed in eight cases and two stents were placed in the remaining two cases. Ability to eat and/or swallow improved and scored 2.4 (score 3 in 5 cases, score 2 in 4 cases, score 1 in 1 case, and score 0 in no case) after stent placement. In two patients with tracheoesophageal fistula and dysphagia, fistula sealing was also achieved and fistula recurrence was not noted until death.

No major complications related to the procedure were encountered and there was no procedure-related mortality. No procedural complications, including esophageal perforation and hemorrhage, occurred, and no patient experienced severe pain at the site of stent placement lasting more than 24 h that needed narcotic analgesics.

Three patients reported a moderate foreign body sensation that was well tolerated and gradually disappeared by the end of the first week after stent placement. The remaining five patients tolerated the stent placement well in the first few days with no further difficulties.

No post-procedural complications, including perforation, hemorrhage, and stent migration, occurred, but recurrence of dysphagia due to circumferential tumor overgrowth at the proximal end of the stent was observed in two cases 22 and 46 days after stent placement, respectively. Reintervention was not performed in these two cases because the patients refused further therapy. Tumor ingrowth did not occur in any case. The mean survival time was 131 ± 77 days (range = 31–259 days).

## Discussion

Obstruction of the esophagus leads to progressive dysphagia, malnutrition, and aspiration pneumonia. Dysphagia is usually the most distressing symptom in patients with inoperable malignancies of the esophagus, necessitating immediate palliation [1, 17, 18].

Esophageal cancer located in the cervical region is uncommon, accounting for 7–10 % of all esophageal cancers [19]. Treatment for a tumor located in this region is different from that for a tumor located in the intrathoracic segment of the esophagus [19, 20].

The cervical esophagus endoscopically is between approximately 15 and 20 cm from the incisor teeth and radiologically projects above the sternoclavicular joint [21]. At that level in which the resting wall tension is high, which is a high-pressure zone, any endoscopic procedure is more problematic and troublesome, even in the presence of normal anatomy, since long and flexible endoscopy of the hypopharynx and upper esophageal sphincter is technically difficult due to the reduced efficacy of insufflation and movements-related swallowing [12, 21]. Therefore, it is relatively difficult to achieve exact placement of the stent in patients with cervical cancer compared to other esophageal regions with direct endoscopy guidance. Large series studies of SEMS placement in cervical region are lacking [11, 12].

In addition, there have been few reports on the placement of esophageal prostheses for cervical lesions because of concerns about the high risk of proximal migration of the stent into the hypopharynx and the intolerable foreign body sensation, severe throat pain, and sudden upper respiratory tract occlusion that may occur [22–24].

Currently, fluoroscopy has become the conventional approach for guiding stent placement. Many studies have reported insertion of stents under radiologic guidance without endoscopic assistance [13, 17, 25]. It is accepted that the cervical esophagus is between the sixth cervical vertebra at the pharyngoesophageal junction and the thoracic inlet at the first thoracic vertebra [21]. However, the location of the cervical esophagus could vary among individuals, and fluoroscopy does not always accurately demonstrate the location of the upper esophageal orifice. Even placement under fluoroscopy has limitations when attempting exact positioning of the stent [21, 26].

Many studies have reported the usefulness of covered SEMSs that were deployed under direct endoscopic visualization and fluoroscopic guidance [14, 18, 23]. Nonetheless, under endoscopic and fluoroscopic guidance, Austin et al. [27] reported unsatisfactory stent positioning for 7 of 30 patients with unresectable esophageal cancer. Lazaraski et al. [8] also reported that the stent was not accurately positioned when deployed in 7 of 89 patients under endoscopic guidance. Misplacement of the stents has occurred frequently and might diminish their therapeutic efficacy.

It is not necessary to be as accurate with the placement of a stent in the thoracic esophagus between the superior margin of the sternum and the inferior tracheal bifurcation because minor misplacement of the stent might not lead to serious complications [11, 12]. However, it is very important to precisely place a stent in the cervical esophagus because stent misplacement may reduce its effectiveness and cause unexpected complications. In patients with cervical esophageal cancer, when the proximal end of the SEMS reaches the upper esophageal orifice, it can cause a swallowing disturbance or serious discomfort or pain in the cervical region [11, 12, 22, 25]. Moreover, the risk of cervical stenting relates to the possibility of proximal misplacement, which shares the danger of sudden upper respiratory tract occlusion [11]. Mcdonald et al. [28] reported that the upper limit of the stent is the fifth cervical vertebra; however, some of their patients complained of a foreign body sensation. Therefore, exact placement of the stent is absolutely critical in patients with cervical esophageal cancer.

In the present study, CT guidance was used in addition to fluoroscopy. To our knowledge, there have been no studies reporting placement of an esophageal stent under CT guidance. The IVR-CT system has the ability to obtain fluoroscopy and thin-slice CT within one session. The cervical anatomy and tumor location can be clearly demonstrated by multidetector CT. We employed CT in the present study because it provides detailed imaging of the soft tissues and surrounding structures of the cervical region within one diagnostic tour, allowing the safest identification of the exact location for stent placement. Furthermore, MPR images provide useful information on the esophageal wall and the extension of tumor invasion. The use of CT guidance has the potential of avoiding stent misplacement.

The Niti-S stent used in the present series becomes shortened at the distal end when it is deployed, so that adjustment of the position of the proximal end of the stent is relatively easy and misplacement can be avoided[14]. On the other hand, the Ultraflex stent is difficult to place exactly because both ends of the stent become shortened when the stent is deployed. Also, because of its double layer configuration with a membrane, migration of the Niti-S stent is less likely [14, 15].

The present results indicate that the Niti-S stent can be placed in the desired position under CT and fluoroscopic guidance. Furthermore, the frequency of procedural and post-procedural complications was almost equal to that of other SEMSs [8, 9, 17].

There are three important limitations to this study. First, this was a small study with a limited number of patients. Therefore, randomized trials comparing the efficacies, risk of complications, and recurrent dysphagia of covered stents of various designs are needed, with particular attention given to stent migration and tumoral and nontumoral tissue overgrowth. Moreover, all procedures were performed by only two experienced radiologists so the results might be biased.

Second, in two patients, overgrowth of the tumor at the proximal end of the stent occurred. In patients in whom the tumor invaded directly below the esophageal orifice, because the SEMS could not cover the proximal end of the tumor, stent use in this region has limitations. Third, CT with fluoroscopy might increase the cost of the procedure compared to an endoscopic approach.

## Conclusions

The present results demonstrate that the Niti-S stent is a safe and effective device for the palliation of dysphagia caused by inoperable or advanced cervical esophageal cancer. The incidence of procedure-related complications is comparable to that of other covered metal stents. The outer wire of the Niti-S stent is likely to reduce stent migration in patients with cervical esophageal cancer. The distal shortening of the stent when it is deployed is also helpful for avoiding proximal misplacement of the stent. In addition, combined multidetector CT and fluoroscopic image guidance provides the correct anatomical location to the operator.
